# Parasitism by endoparasitoid wasps alters the internal but not the external microbiome in host caterpillars

**DOI:** 10.1186/s42523-021-00135-y

**Published:** 2021-10-15

**Authors:** Gabriele Gloder, Mitchel E. Bourne, Christel Verreth, Liesbet Wilberts, Sofie Bossaert, Sam Crauwels, Marcel Dicke, Erik H. Poelman, Hans Jacquemyn, Bart Lievens

**Affiliations:** 1 CMPG Laboratory for Process Microbial Ecology and Bioinspirational Management (PME&BIM), Department M2S, KU Leuven, Willem De Croylaan 46, 3001 Leuven, Belgium; 2 Leuven Plant Institute (LPI), KU Leuven, 3001 Leuven, Belgium; 3 Laboratory of Entomology, Wageningen University, Droevendaalsesteeg 1, 6708 PB Wageningen, The Netherlands; 4 Laboratory of Plant Conservation and Population Biology, Biology Department, KU Leuven, Kasteelpark Arenberg 31, 3001 Leuven, Belgium

**Keywords:** *Cotesia glomerata*, Microbial community, Parasitism, *Pieris brassicae*, Trophic interactions, *Wolbachia*

## Abstract

**Background:**

The microbiome of many insects consists of a diverse community of microorganisms that can play critical roles in the functioning and overall health of their hosts. Although the microbial communities of insects have been studied thoroughly over the past decade, little is still known about how biotic interactions affect the microbial community structure in and on the bodies of insects. In insects that are attacked by parasites or parasitoids, it can be expected that the microbiome of the host insect is affected by the presence of these parasitic organisms that develop in close association with their host. In this study, we used high-throughput amplicon sequencing targeting both bacteria and fungi to test the hypothesis that parasitism by the endoparasitoid *Cotesia glomerata* affected the microbiome of its host *Pieris brassicae*. Healthy and parasitized caterpillars were collected from both natural populations and a laboratory culture.

**Results:**

Significant differences in bacterial community structure were found between field-collected caterpillars and laboratory-reared caterpillars, and between the external and the internal microbiome of the caterpillars. Parasitism significantly altered the internal microbiome of caterpillars, but not the external microbiome. The internal microbiome of all parasitized caterpillars and of the parasitoid larvae in the caterpillar hosts was dominated by a *Wolbachia* strain, which was completely absent in healthy caterpillars, suggesting that the strain was transferred to the caterpillars during oviposition by the parasitoids.

**Conclusion:**

We conclude that biotic interactions such as parasitism have pronounced effects on the microbiome of an insect host and possibly affect interactions with higher-order insects.

**Supplementary Information:**

The online version contains supplementary material available at 10.1186/s42523-021-00135-y.

## Introduction

Although the past decade has witnessed an enormous rise in studies characterizing the microbiome of insects [1, 2], little is still known about how microbial communities assemble in and on the bodies of insects. Although not necessarily true for all insects [3, 4], microorganisms can play a critical role in the fitness and overall health of insects [5–8] or provide protection against pathogens and support detoxification of pesticides or harmful plant secondary metabolites [9–11]. Gut microbial communities often deliver metabolic benefits to their hosts by the production of vitamins and providing digestive enzymes that improve nutrient uptake [1, 12]. The composition of insect gut microbial communities varies extensively between insect taxa and it is associated with the environment, diet, developmental stage, and phylogeny of the host [1, 13, 14]. Several hundreds of bacterial phylotypes have been described in termites [15, 16] and over a few tens in Lepidoptera [17, 18], while there is an almost complete absence of bacteria in aphid guts [19]. In contrast to bacteria, only very little is known about fungi, possibly because fungi are particularly associated with insects feeding on wood or detritus [1, 20]. Similarly, the external surfaces of animals, including the exoskeleton of insects, are commonly colonized by microorganisms [21]. Although only little is known about their ecological role, external microbes are assumed to play a major role in body odour [22, 23] and contribute to increased defence against predators and survival [24]. Besides, by altering body odours external microbes may also signal the presence of suitable prey or hosts [25, 26].

Recent studies that have compared the internal and external component of the insect microbiome revealed higher diversity of the external microbial community [27–29]. In general, insect guts are colonized by bacteria ingested with food that are able to survive and thrive in the gut [27, 30]. Furthermore, in most insects a substantial part of the internal microbiome consists of specialized gut symbionts that are obtained by vertical transmission, resulting in a gut microbiome consisting of several resident “core” microbiota [31]. In contrast, the external microbiome is often composed of microorganisms that commonly occur in the environment, and has been shown to vary significantly with geographic location and habitat [29], suggesting that local environmental conditions and local availability of microbes strongly determine the external microbial community on insects.

In insects that are attacked by parasites or parasitoids (i.e. insects whose larvae live as parasites in other insects and eventually kill their hosts), it can be expected that the microbiome of the host insect is to some extent affected by the presence of these parasitic organisms that develop in intimate association with their host. Furthermore, insect parasitoids harbour their own microbial communities, including symbionts, that may be transferred to the next generation and also affect the host microbiome [21, 32]. Parasites like helminths and protozoa residing in the insect gut have been shown to alter the composition of the gut microbiome, and may thereby strongly impact host immunity and gut homeostasis [33]. Likewise, larvae of endoparasitoids that feed on host's tissues and/or hemolymph may impact the internal microbial community of host insects, while having less or no impact on the external community. However, at present very little is known about how parasites or parasitoids affect the microbiome of their host insects (but see [34]).

In this study, we used high-throughput amplicon sequencing targeting both bacteria and fungi to test the hypothesis that parasitism by endoparasitoids affects the microbiome of host insects. Specifically, we compared the internal and external microbiome of healthy and parasitized caterpillars of the large cabbage white *Pieris brassicae* (Lepidoptera: Pieridae) and one of its main parasitoids, *Cotesia glomerata* (Hymenoptera: Braconidae). Additionally, we assessed the internal and external microbiome of the developing parasitoid larvae in parasitized caterpillars. Caterpillars were collected from both natural populations and a laboratory culture to identify whether parasitoids consistently alter the microbiome of host caterpillars across origin of the caterpillars.

## Results

### Bacterial and fungal diversity

**Fig. 1:**
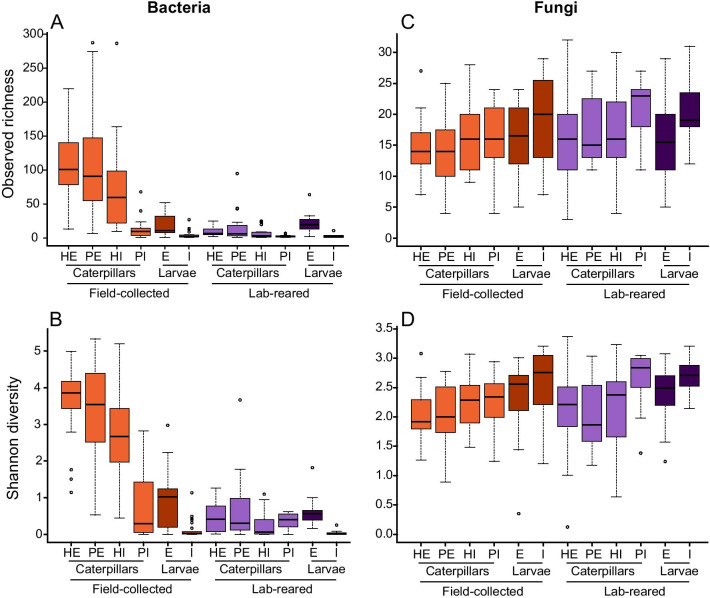
Boxplots showing alpha diversity comparisons of the different caterpillars (*Pieris brassicae*) and parasitoid larvae (*Cotesia glomerata*) samples studied. Samples were divided in different subgroups according to habitat, health status and origin for caterpillars, and habitat and origin for parasitoid larvae. The upper and lower whiskers correspond to the first and third quartiles, with the bar in the middle marking the median value. Alpha diversity was measured by the number of observed of (z)OTUs (top panels) and Shannon index (bottom panels) for bacteria (**A**, **B**) and fungi (**C**, **D**). Abbreviations used: H = healthy; P = parasitized; E = external; and I = internal

After quality filtering, removal of rare sequences and rarefying, a total of 4,287 bacterial zOTUs and 707 fungal OTUs were retained for further analysis (Additional file 1: Table S1 and S2). Rarefaction curves approached saturation, indicating that our sequencing depth was sufficient to cover the microbial diversity (Additional file 2: Fig. S1). Alpha diversity comparisons of the bacterial communities on and in the caterpillars revealed significant (*F*_1,196_ = 122.370; *p* < 0.001) differences between field-collected and lab-reared caterpillars (Fig. 1A, B; Additional file 1: Table S3). Overall, on average 75.6 (range: 1–288) bacterial zOTUs were associated with field-collected caterpillars, while only 8.9 zOTUs (range: 1–95) were found in the lab-reared caterpillars. Shannon diversity was also significantly higher (*F*_1,196_ = 309.586; *p* < 0.001) in the field-collected caterpillars compared to the lab-reared caterpillars (mean Shannon diversity: 2.7 and 0.4, respectively) (Fig. 1B; Additional file 1: Table S3 and S4). The external microbiome of the caterpillars was also significantly (*F*_1,196_ = 88.817; *p* < 0.001) more diverse in terms of bacteria than the internal microbiome (Fig. 1A, B; Additional File 1: Table S3). This was especially the case for field-collected caterpillars, having a mean zOTU richness of 107.3 (range: 7–288) and a mean Shannon diversity of 3.5 (range: 0.5–5.3) for the external samples, compared to 43.9 (range:1–287) and 1.8 (range: 0–5.2) for the internal samples, respectively (Fig. 1A, B; Additional file 1: Table S4). Bacterial communities of the external microbiome of the parasitoid larvae were also more diverse (*F*_1,81_ = 42.610; *p* < 0.001) than those of the internal microbiome, but differences in diversity between parasitoid larvae from field-collected and lab-reared caterpillars were small (Fig. 1A, B). On average, 20.6 zOTUs (range: 1–64) were found on the outside of the parasitoid larvae, while 3.9 zOTUs were found inside (range: 1–27) (Fig. 1A, B; Additional file 1: Table S4).

Fungal communities showed less variation in diversity compared to bacteria, with no or little variation between field-collected and lab-reared caterpillars and between the external and internal microbiome (Fig. 1C, D; Additional file 1: Table S3). On average, 15 fungal OTUs (range: 3–32) were found in the external microbiome of the caterpillars, while the internal microbiome comprised on average 17 fungal OTUs (range: 4–30) with only little variation between parasitized and healthy caterpillars and between field-collected and lab-reared individuals (Fig. 1C; Additional file 1: Table S5). The average Shannon diversity of the fungal communities from the external compartment of the caterpillars was 2.0 (range: 0.1–3.4), and 2.3 for the internal compartment (range: 0.6–3.2) (Fig. 1D; Additional file 1: Table S5). Similar values were obtained for the parasitoid larvae (Fig. 1C, D; Additional file 1: Table S5).

**Fig. 2:**
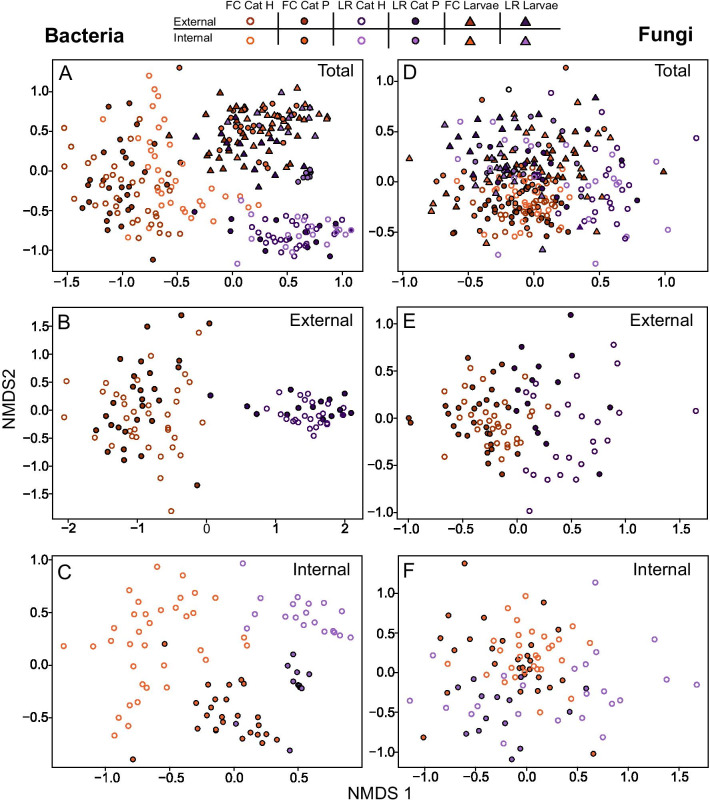
Non-metric multidimensional scaling (NMDS) ordination plots based on Bray–Curtis distances of Hellinger-transformed relative abundance data of the bacterial (**A**–**C**) and fungal communities (**D**–**F**) of the different caterpillars (*Pieris brassicae*) and parasitoid larvae (*Cotesia glomerata*) samples studied. Results for all samples are shown in **A** (stress = 0.181) and **D** (stress = 0.282). Results for the external samples are shown in **B** (stress = 0.170) and **E** (stress = 0.239), and for the internal samples in **C** (stress = 0.181) and **F** (stress = 0.266). Abbreviations used: Cat = caterpillars; H = healthy; P = parasitized; FC = field-collected; and LR = lab-reared

Non-metric multidimensional scaling (NMDS) ordination of the Bray–Curtis distances of Hellinger-transformed relative abundance data of the bacterial communities revealed clear differences between individuals of field-collected and lab-reared populations and between external and internal samples from healthy and parasitized caterpillars. While the external samples of healthy and parasitized caterpillars grouped together (Fig. 2B), the corresponding internal samples were clearly separated (Fig. 2C). NMDS ordination also separated field-collected and lab-reared caterpillars (Fig. 2A–C). In contrast, samples from the parasitoid larvae (both internal and external microbiomes) clustered together, along with the internal samples of the parasitized caterpillars (Fig. 2A). Nonparametric multivariate analysis of variance showed significant differences in bacterial community composition between caterpillars from natural populations and those reared in the laboratory (*F*_1,196_ = 82.136; *p* < 0.001), and between healthy and parasitized caterpillars (*F*_1,196_ = 15.839; *p* < 0.001) (Table 1). Significance of the interaction term was low (*F*_1,196_ = 2.244; *p* = 0.045), indicating that effects of parasitism were not strongly affected by habitat. There was also a strong significant difference between the internal and external microbiome (*F*_1,196_ = 16.950; *p* < 0.001), and this difference depended strongly on the health status (*F*_1,196_ = 15.337; *p* < 0.001) and habitat (*F*_1,196_ = 5.981; *p* < 0.001) of the caterpillars. Likewise, there was a three-way interaction effect, but significance was low (*F*_1,196_ = 2.122; *p* = 0.045) (Table 1). A significant difference was found between bacterial communities from the internal microbiome of healthy and parasitized caterpillars (*F*_1,98_ = 37.405; *p* < 0.001), while no significant difference was found for the external microbiome (*F*_1,98_ = 0.891; *p* = 0.476) (Table 1). For fungi the NMDS did not show such clear patterns as for bacteria, but fungal communities from field-collected caterpillars also diverged from the lab-reared caterpillars (*F*_1,183_ = 17.987; *p* < 0.001) (Table 1).

**Table 1:** Results of PERMANOVA on bacterial and fungal community compositions (caterpillars only)^a^

	Bacteria						Fungi					
	E and I (*n* = 204)		E (*n* = 102)		I (*n* = 102)		E and I (*n* = 191)		E (*n* = 98)		I (*n* = 93)	
	*F*	*p*	*F*	*p*	*F*	*p*	*F*	*p*	*F*	*p*	*F*	*p*
Habitat	82.136	< 0.001	42.382	< 0.001	46.534	< 0.001	17.987	< 0.001	14.972	< 0.001	5.635	< 0.001
Health status	15.839	< 0.001	0.891	0.476	37.405	< 0.001	3.029	< 0.001	2.0027	0.019	2.093	0.003
Habitat: Health status	2.244	0.045	0.666	0.836	4.422	0.003	2.663	0.002	1.715	0.044	2.189	0.003
Origin	16.950	< 0.001					3.827	< 0.001				
Origin: Health status	15.337	< 0.001					1.073	0.357				
Origin: Habitat	5.981	< 0.001					2.074	0.008				
Habitat: Origin: Health status	2.122	0.045					1.278	0.147				

^a^E, external; I, internal

### Bacterial and fungal density

**Fig. 3:**
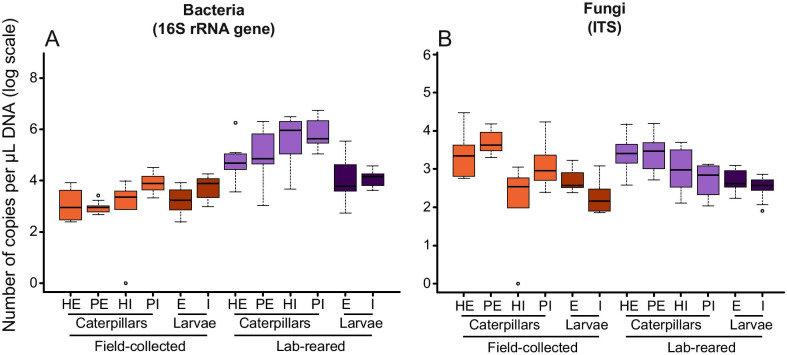
Boxplots showing the numbers of bacterial 16S rRNA gene (**A**) and fungal ITS copies (**B**) for the different caterpillars (*Pieris brassicae*) and parasitoid larvae (*Cotesia glomerata*) samples studied. Samples were divided in different subgroups according to habitat, organism, health status and origin for caterpillars, and habitat and origin for parasitoid larvae. The upper and lower whiskers correspond to the first and third quartiles, with the bar in the middle marking the median value. For each subgroup, ten random samples were analyzed. Abbreviations used: H = healthy; P = parasitized; E = external; and I = internal

Significantly higher amounts of bacteria (*F*_1,72_ = 136.116 *p* < 0.001) were found on and in lab-reared caterpillars compared to the caterpillars from the field. No significant differences were observed between healthy and parasitized caterpillars, nor between internal and external samples (Fig. 3A; Additional file 1: Table S3). On average, the internal and external microbiome of lab-reared caterpillars contained 1.23 × 10^6^ and 3.30 × 10^5^ 16S rRNA gene copy numbers per μL of DNA, respectively, while this was 7.51 × 10^3^ and 1.74 × 10^3^ for the field-collected caterpillars, respectively (Additional file 1: Table S6). By contrast, no differences were found in bacterial 16S rRNA gene copy numbers between parasitoid larvae from field-collected and lab-reared caterpillar populations (Fig. 3A; Additional file 1: Table S6). Fungal densities were highly comparable between field-collected and lab-reared caterpillars (Fig. 3B), having an overall average of 1.70 × 10^3^ and 1.14 × 10^3^ ITS copies per µL DNA for the internal compartment and 5.57 × 10^3^ and 3.94 × 10^3^ for the external compartment of the microbiome, respectively (Additional file 1: Table S7). Similar fungal densities were also found in the parasitoid larvae. Among the field-collected caterpillars, the internal microbiome contained fewer fungal ITS copy numbers in healthy caterpillars (mean: 4.86 × 10^2^) than in parasitized caterpillars (mean: 2.67 × 10^3^). There was no difference in the number of ITS copy numbers between the external microbiome of healthy (mean: 5.02 × 10^3^) and parasitized caterpillars (mean: 6.12 × 10^3^) (Fig. 3B) (Additional file 1: Table S7).

### Microbial community composition

**Fig. 4:**
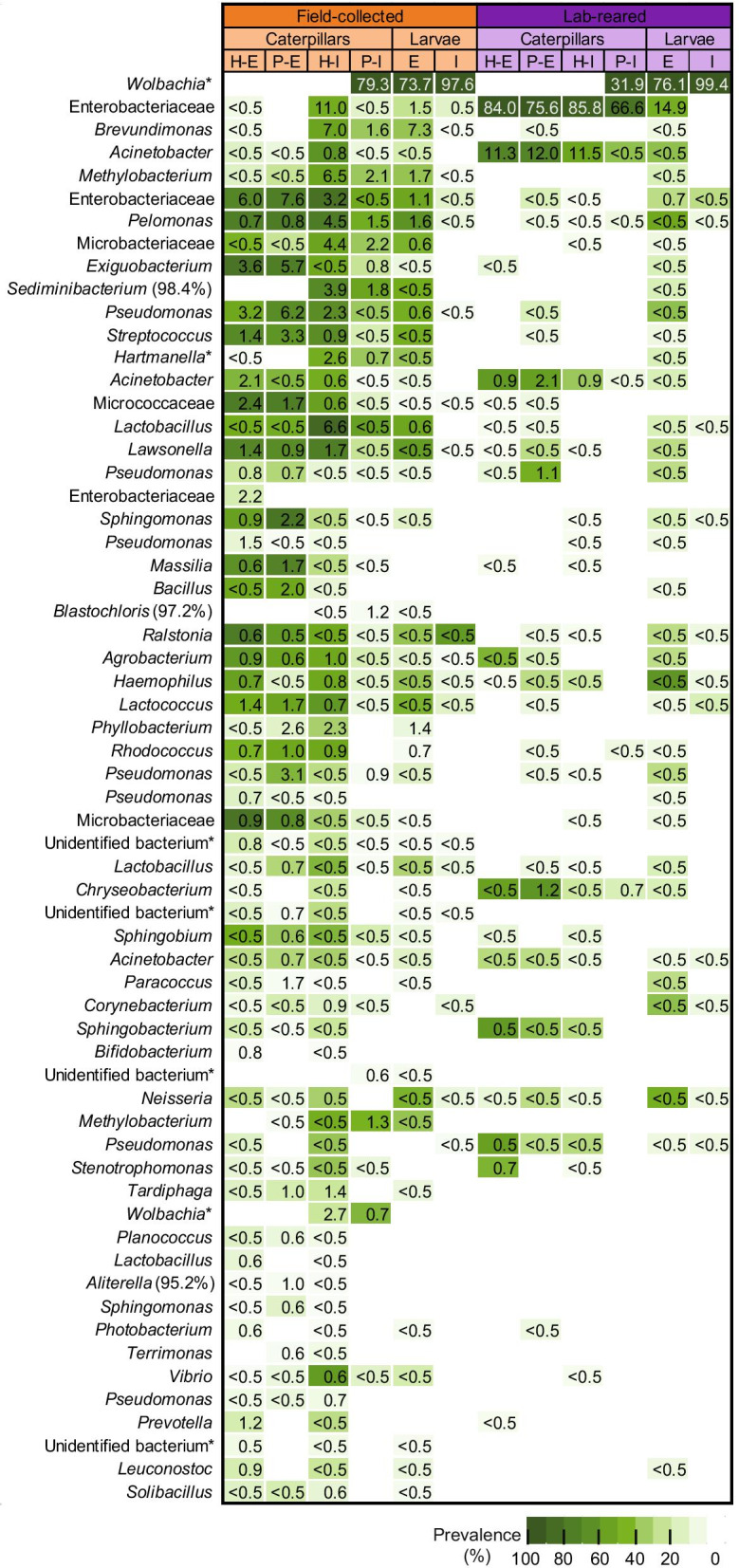
Bacterial community profiles of the different caterpillars (*Pieris brassicae*) and parasitoid larvae (*Cotesia glomerata*) samples studied. Bacterial taxa represent the most prevalent taxa in the different subgroups based on origin and health status for caterpillars and origin for parasitoid larvae (present at a mean relative abundance > 0.5% in at least one subgroup). For each zOTU, the average relative abundance for each subgroup is given in the box as a percentage, whereas the color indicates prevalence (white is absent). zOTUs are identified by a BLAST search against type materials in GenBank. When no significant similarity was found with type materials, the BLAST analysis was performed against entire GenBank (indicated with and asterisk). Identifications were performed at genus level; when identical scores were obtained for different genera, identifications were performed at family level. When identity percentages were lower than 99%, the percentage of sequence identity with the GenBank entry is given between brackets. Hits with uncultured bacteria are indicated as unidentified bacterium. Abbreviations used: H = healthy; P = parasitized; E = external; and I = internal

Bacteria found in and on the caterpillars investigated represented several environmental and insect-associated species belonging to diverse phyla, among which the most abundant were Proteobacteria, Firmicutes and Actinobacteria (Additional file 1: Table S1). Irrespective of health status, the external microbiome of field-collected caterpillars was composed of diverse bacteria from different phyla occurring at relatively low abundances. By contrast, lab-reared insects showed bacterial communities (both external and internal) that were characterized by two highly abundant zOTUs, i.e. an unidentified Enterobacteriaceae member (zOTU2) and *Acinetobacter* sp. (zOTU5). zOTU2 was detected in almost every lab-reared caterpillar at an overall average relative abundance of 79.6% (calculated based on the entire dataset), while zOTU5 occurred in about half of the caterpillars at an overall average relative abundance of 9.4%. In contrast, both zOTUs were absent or occurred at lower relative densities in the natural populations (Fig. 4). Interestingly, the internal samples of all parasitized caterpillars studied, including both field-collected and lab-reared caterpillars, as well as all samples taken from the parasitoid larvae were dominated by one particular bacterium (zOTU1), the insect symbiont *Wolbachia pipientis* (Fig. 4). Relative abundance of this symbiont ranged from 3.2% up to 97.6% (mean: 62.7%) in the internal microbiome of parasitized caterpillars, with a lower relative abundance in the lab-reared insects (mean: 31.9%) compared to field-collected caterpillars (mean: 79.3%). Further, the bacterium was found in and on all parasitoid larvae. For parasitoid larvae from field-collected caterpillars, it occurred at a mean relative abundance of 73.7% (4.8–100%) in the external microbiome and 97.6% (79.6–100%) in the internal microbiome. Similarly, in parasitoid larvae from lab-reared insects it was present at a relative abundance of 76.1% (1.9–97.7%) and 99.4% (95.8–100%) in the external and internal samples, respectively. In addition to *Wolbachia*, the external samples of the parasitoid larvae contained a huge variety of other microorganisms, which were, especially for the field-collected caterpillars, also found in the internal microbiome of parasitized and unparasitized caterpillars (Fig. 4). In contrast, the *Wolbachia* zOTU was completely absent in samples from healthy caterpillars, and was also not found in the external samples from parasitized caterpillars (Fig. 4), as was also confirmed by a specific PCR targeting *Wolbachia* DNA (Additional file 1: Table S8).

The fungal community composition was homogeneous among all samples studied, with fewer notable differences between field-collected and lab-reared caterpillars (Additional file 2: Fig. S2). Fungal communities were dominated by two *Alternaria* species, OTU6 and OTU8, and one *Sporobolomyces* sp. (OTU10), occurring in 74.4, 83.9 and 73.3% of all samples studied, respectively. Additionally, *Malassezia* sp*.* (OTU16) was commonly detected and occurred in 37.4% of the samples studied (Additional file 1: Table S2).

## Discussion

Although the microbiome of insects has been studied thoroughly over the past decade [35, 36], including Lepidoptera [17, 37], little is still known about how microbial communities assemble in and on the bodies of insects, and to which extent insect microbiomes are affected by the presence of parasites [38] or parasitoids (but see [34]). Our taxonomic analysis revealed that the bacterial microbiome of *P. brassicae* caterpillars was mainly composed of Proteobacteria, Firmicutes and Actinobacteria. These phyla represent the most common phyla in lepidopteran species, including *Pieris* spp. [18, 34, 39–41]. In contrast to other studies that found very low bacterial abundances in *P. brassicae* caterpillars [42], estimation of the bacterial abundance by qPCR suggested higher bacterial densities in our samples. This was confirmed by plating a selection of samples on trypticase soy agar supplemented with 0.5 g/L cycloheximide (up to10^4^ bacterial colony forming units (cfu) per specimen) (Additional file 1: Table S9). Members of the Ascomycota and Basidiomycota were the most common fungi found in our dataset, including several environmental fungi that commonly occur on cultivated plants like *Alternaria*, *Cladosporium* and *Sporobolomyces*. Fungal symbionts of insects have been mostly studied in insects feeding on wood or detritus [1, 15], but have been recently studied in Lepidoptera as well [43]. Although fungi may contribute to the provision of nutrients and regulation of host defenses in insects, the exact functions that most fungi play in associations with insects are yet to be discovered [2].

Our results further showed that caterpillars from natural populations harbour a more diverse and evenly distributed bacterial microbiome than lab-reared caterpillars, both at the outside and the inside of the insect. While on average 75.6 bacterial zOTUs were found in the field-collected caterpillars, the microbiome of lab-reared caterpillars comprised an average of only 8.9 zOTUs. Two bacteria were dominant in and on the bodies of lab-reared caterpillars, while they only sporadically occurred in natural populations. These bacteria were identified as an *Acinetobacter* species and a member of the family Enterobacteriaceae. *Acinetobacter* species and Enterobacteriaceae are ubiquitous in nature, and occur in diverse habitats, including soil, plants and insect guts [1, 37, 41, 44, 45]. The reason why they occur more abundantly in lab-reared insects compared to wild-collected insects is not yet clear, but similar patterns in diverging microbiome composition between natural and lab-reared insect populations have been observed in other caterpillars (*Spodoptera* spp.) [46], house flies (*Musca domestica*) [29], fruit flies (*Drosophila* spp.) [27] and leafhoppers (*Psammotettix alienus*) [47]. Factors like diet and ecological and environmental differences between natural habitats and artificial rearing environments seem to play a major role, while parental effects are less important [1, 14, 27, 41, 46] Larvae of most butterfly species largely mirror the bacterial community composition of their diets, suggesting passive acquisition of their bacterial gut inhabitants through food ingestion rather than active selection [41]. Indeed, previous studies have shown that caterpillars lack resident gut symbionts, and mainly harbor transient environmental microorganisms that are present on host plants, including microorganisms that originate from the soil and are transferred through the plant [48, 49]. The stable environmental conditions when rearing insects in the laboratory or the limited pool of bacteria present in the rearing facilities may have favored the growth of particular fast-growing species that outcompeted or reduced the growth of other species. This may also explain the higher bacterial concentrations in samples from lab-reared caterpillars compared to field-collected caterpillars. While less clear, also for fungi differences were found in fungal diversity between field-collected and lab-reared caterpillar populations. Nevertheless, it has to be noted that fungal density was rather low in our samples; in general ITS copy numbers varied between 10^2^ and 10^4^ copies per µL DNA. Given the fact that fungi can possess more than 100 ITS copies per genome [50], these values thus represent low densities, which was also confirmed by plating a subset of samples on yeast potato dextrose agar with 0.5 g/L chloramphenicol (up to 10^2^ fungal cfu per specimen (Additional file 1: Table S9)).

The external bacterial microbiome of the caterpillars was significantly more diverse than its internal counterpart. Our results show that parasitism altered the internal microbiome of caterpillars, but not the external microbiome. The internal samples of all parasitized caterpillars as well as all samples taken from the parasitoid larvae were dominated by one particular bacterial strain, the insect symbiont *Wolbachia pipientis* (zOTU1)*,* while it was completely absent in healthy caterpillars. In some parasitoid larvae *W. pipientis* was the only bacterium detected. This pattern was present in both wild and lab-reared caterpillars, although its relative abundance was higher in parasitized caterpillars from the field (79.3%) compared to parasitized caterpillars from the lab (31.9%). The factors driving this difference in relative abundance are not yet clear, but it may be possible that caterpillars reared in the laboratory and collected in the field have developed different immune responses to *Wolbachia* [51]. Also, the microbiome of lab-reared caterpillars was strongly dominated by a member of the family Enterobacteriaceae, which occurred to a much lesser extent in field-collected caterpillars, and which may have inhibited the *Wolbachia* strain from excessive reproduction in the lab-reared caterpillars. Furthermore, caution must be taken with the biological interpretation of relative abundance data, since inter-sample differences in cell density are not considered [52].

*Wolbachia* is a genus of well-studied intracellular endosymbionts that are commonly found in arthropods and that are able to manipulate host reproduction to favor its own maternal transmission [53, 54]. However, *Wolbachia* is often mutualistic for many insects, as it provides its host resistance against viruses, insecticides or plant defenses, and contributes to nutritional provisioning [55, 56]. *Wolbachia* is estimated to be present in about 80% of lepidopteran species, including species belonging to the Pieridae family [57]. Interestingly, in addition to the *Wolbachia* strain dominating parasitized *P. brassicae* caterpillars, we also found another *Wolbachia* strain (zOTU101) in a few healthy (four samples) and parasitized field-collected caterpillars (8 samples), while it did not occur in any lab-reared caterpillar, suggesting a rather limited distribution of this strain (Fig. 4; Additional file 1: Table S1). At the nucleotide level, both *Wolbachia* strains shared 95.6% 16S rRNA gene sequence identity on a total of 248 bp. *Wolbachia* is also commonly reported in parasitoids [58], including *C. glomerata* [32, 59]. PCR analyses targeting the 16S rRNA gene of *Wolbachia* confirmed its presence in the *C. glomerata* laboratory culture that was used to infect the lab-reared caterpillars in this study (Additional file 1: Table S8). Since it was not found in healthy caterpillars, but was highly present in the internal compartment of parasitized caterpillars as well as in the parasitoid larvae and adults, it is reasonable to assume that the parasitoids transferred *Wolbachia* into the caterpillars during oviposition after which it established and replicated, explaining its high relative abundance in parasitized caterpillars. This is in line with previous studies showing that parasitoids may transfer *Wolbachia* into their host during oviposition, as seen in whiteflies [60]. However, surprisingly *Wolbachia*, being an intracellular bacterium, was also abundantly found on the outside of the parasitoid larvae. One plausible explanation may be that *Wolbachia*-containing host tissues were damaged by larval feeding or during the dissection leading to contamination of the outside of the parasitoid larvae. Likewise, tissue damage and/or gut disruption may explain the high microbial diversity in the external parasitoid samples and explain why they represented a community similar to the internal host samples. Further research is needed to exclude this scenario.

The presence of *Wolbachia* in adult parasitoids could have a positive effect on the wasps by enhancing host-searching ability and oviposition frequency [61]. On the other hand, it has also been suggested that *Wolbachia* can have negative effects on parasitoid populations as it can increase the susceptibility to hyperparasitism by hyperparasitoids, i.e. parasitic wasps that attack the larvae and pupae of primary parasitoids [62, 63]. Hyperparasitoids strongly rely on herbivore-induced plant volatiles (HIPVs) to locate potential hosts [64, 65], but also use other cues such as changes in the body odors of parasitized herbivores to locate their host from a short distance [66]. Although the underlying mechanisms are still unclear, it can be hypothesized that microorganisms may be involved in mediating body odor changes [67], as was recently demonstrated for honey bees [68], and/or may act synergistically with other agents like polyDNAvirus and venom affecting HIPV emission and revealing the presence of parasitoid hosts to its hyperparasitoids [69]. Whether and to which extent *Wolbachia* is involved in this process requires further research.

## Conclusions

Together, our results show that the microbiome of caterpillars from natural populations harbored a much more diverse bacterial microbiome than lab-reared caterpillars. The external microbiome of the caterpillars was also significantly more diverse than its internal counterpart. Fungal communities were less diverse and showed less variation. Our results also clearly show that parasitism significantly altered the internal microbiome of the caterpillars, but not the external microbiome. The internal microbiome of all parasitized caterpillars and of the parasitoid larvae was dominated by a *Wolbachia* strain, while this bacterium was completely absent in healthy caterpillars. Further research is needed to elucidate the possible role of this endosymbiont in the interaction between the host caterpillar, the parasitoid, and higher trophic levels.

## Materials and methods

### Study system

In this study, caterpillars of the large cabbage white *Pieris brassicae* (Lepidoptera: Pieridae) and one of its main parasitoids, *Cotesia glomerata* (Hymenoptera: Braconidae), were used as study organisms. *Pieris brassicae* is an important cosmopolitan pest species of many crops belonging to the family Brassicaceae such as cabbage, cauliflower, brussels sprouts and rape. *Cotesia glomerata* is a gregarious koinobiont wasp that parasitizes a wide range of caterpillars of pierid butterflies, but *P. brassicae* and *Pieris rapae* are its main hosts. The wasp lays approximately 20–40 eggs inside first or second instar caterpillars where the larvae will hatch and consume the body from the inside, while the caterpillars are still alive and continue feeding themselves. Typically, after 15 to 20 days the parasitoid larvae emerge from their caterpillar host, which ultimately kills the caterpillar [70].

### Sample collection

A total of 102 fifth-instar caterpillars of *P. brassicae* were used in this study, including 59 non-parasitized caterpillars and 43 caterpillars parasitized by *C. glomerata* (Additional file 1: Table S10). Among these, 63 caterpillars (35 non-parasitized and 28 parasitized) were collected from the field. Thirty-nine lab-reared individuals (24 non-parasitized and 15 parasitized) were included for the sake of comparison. Field-collected individuals were obtained between July and September 2019 from three organic farms growing cauliflower (*Brassica oleracea* L. var. *botrytis*) (Field 1 and 2, both located in Bornem, Belgium) or white cabbage (*Brassica oleracea* L. var. *capitata*) (Field 3, Randwijk, The Netherlands). To minimize collection of sibling larvae, each field-collected caterpillar was retrieved from a different plant, and sampling was performed over a three months period.

With regard to the lab-reared insects, lab cultures from *P. brassicae* and *C. glomerata* were used that both originated from agricultural fields near Wageningen University, The Netherlands. The *P. brassicae* culture was reared and maintained on Brussels sprouts plants (*Brassica oleracea* L. var. *gemmifera* cv. Cyrus) in a large cage in a greenhouse compartment (21 ± 1 °C, 25–35% RH, 16:8 h light/dark). Male and female butterflies were allowed to freely mate in the cage and lay their eggs on different plants. Adults were fed with a saturated sugar solution. *Cotesia. glomerata* was reared in another cage on *P. brassicae* under the same conditions. When *C. glomerata* larvae had pupated, pupae were collected and transferred to a smaller cage with no plants and emerged parasitoids were provided with honey and water until they were used in the experiments. When *P. brassicae* larvae had hatched from our rearing, multiple cohorts of early first instar larvae from different egg-clutches were collected. To minimize a priori variation between healthy and parasitized caterpillars, for each egg-clutch hatchlings were subjected to two treatments: half of the caterpillars were parasitized by *C. glomerata* and the other half was left untreated*.* In order to parasitize the larvae, caterpillars were put into a clean plastic cage (one cage for hatchlings from the same egg-clutch) with mated *C. glomerata* females and exposed to parasitism for five minutes. For each egg-clutch both groups of caterpillars were then put on Brussels sprouts in two separate cages (one cage for parasitized caterpillars and one for non-parasitized caterpillars; different cages were used for hatchlings from different egg-clutches) within the same greenhouse compartment, until the caterpillars were collected for further analysis. Caterpillars used in the experiment were randomly picked from each cage, and represented individuals from different egg-clutches. All caterpillars were collected using a pair of tweezers that was sterilized by applying 70% ethanol before the collection of each caterpillar. Additionally, gloves were worn that were sterilized with ethanol before a caterpillar was collected. When caterpillars were collected, they were placed individually in empty plastic sterile containers (12 cm diameter; 5 cm height) with a pierced lid, which in case of field-collected caterpillars were transported to the laboratory in a cooling box. Subsequently, caterpillars were left starving overnight at room temperature to allow the insects to empty their gut content, while minimizing contact with their own frass.

### Microbiome sampling

**Fig. 5:**
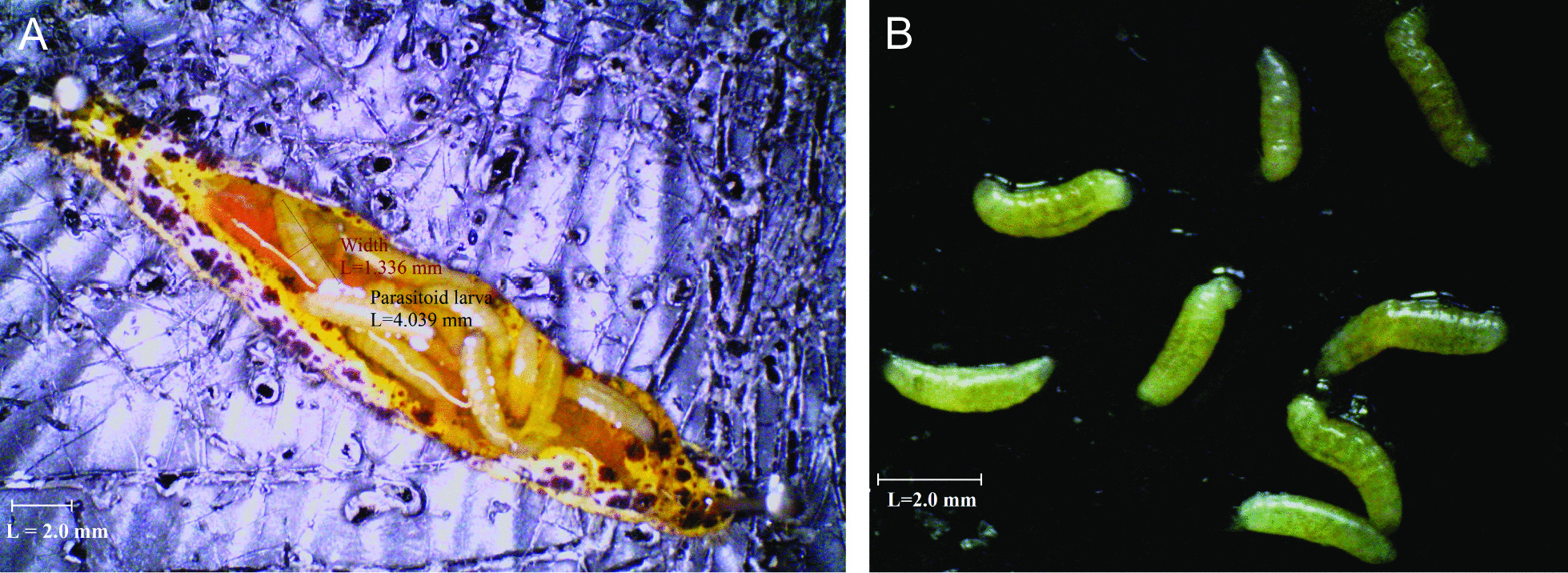
**A** Dissection of *Cotesia glomerata* larvae from *Pieris brassicae* caterpillars. Each caterpillar contained between 20 and 40 late stage parasitoid larvae that were close to egression (ca. 3–4 mm). **B** Close-up picture of some larvae

Both the microbiota associated with the surface of the cuticle and the interior of the insect body were sampled. The external microbiota of the caterpillars were obtained by putting each caterpillar in a 2 mL microcentrifuge tube containing 1 mL of phosphate-buffered saline with 0.01% Tween80 (PBS-T), and vortexing it for 20 s. The washing solution was then used as a sample from the external microbiome. Subsequently, to remove potential residual external microbes, the caterpillar was placed into another tube containing 1 mL of sodium hypochlorite (2.5%) and vortexed again for 20 s, followed by two final washing steps in PBS-T [29]. Application of 2.5% bleach has been shown to be very effective in removing externally contaminating DNA [71, 72]. Each caterpillar was then dissected under sterile conditions to confirm whether or not the caterpillars had been parasitized, and collect the parasitoid larvae. To this end, caterpillars were pinned to a sterile dissection plate with flame-sterilized needles (one in the head and one at the posterior end) and cut open along the entire length of the caterpillar. Next, after opening the insect and pinning both sides with two additional needles, some drops of sterile water were applied on the dissected body in order to ease isolation of the parasitoid larvae (parasitoid larvae float in water). To avoid contamination of the parasitoid larvae with host microbes, the dissection was performed very carefully, aiming to not disrupt the host gut or any other tissues. For each parasitized caterpillar, all parasitoid larvae found inside the caterpillar’s body were removed using a sterilized pair of tweezers and put together in a clean microcentrifuge tube. In general, between 20 and 40 late stage parasitoid larvae (close to egression (ca. 3–4 mm)) were retrieved from all parasitized caterpillars (Fig. 5). The whole body remainder of the caterpillars was then homogenized with a Bead Ruptor Elite (Omni international, Kennesaw, USA) in 1 mL PBS-T and a mixture of glass beads of different sizes (three beads of 2 mm and two beads of 5 mm in diameter) using two cycles of 10 s at a speed of 5.5 m/s with a 10 s break in between. The resulting homogenate was used as a sample reflecting the internal microbiota. Additionally, for the parasitized caterpillars, the external and internal microbiomes from the pool of parasitoid larvae were also collected following the same protocol, with the exception for the working volume used (300 µL instead of 1 mL). PCR screening [73] of the parasitoid larvae confirmed that all parasitized field-collected caterpillars were infested with *C. glomerata*, which is in agreement with the high number of parasitoid larvae found in the caterpillars. Although there are also other parasitic wasps than *C. glomerata* that can attack *P. brassicae*, most of them (if not all of them) are solitary wasps injecting only one egg per caterpillar [74]. Moreover, in contrast to other parasitoids such as *Cotesia rubecula* and *Hyposoter ebeninus*, that interrupt development of *P. brassicae* caterpillars at the 3^rd^ instar, caterpillars parasitized by *C. glomerata* can develop until the 5^th^ larval instar [66], indicating that the parasitized caterpillars collected from the field were only parasitized by *C. glomerata*.

### DNA extraction and molecular analysis

Genomic DNA was isolated from all external and internal samples (300 µL) using the PowerPro Soil Kit (Qiagen, Hilden, Germany) following the manufacturer’s instructions, with one modification: in the second step of the protocol the use of a vortex adapter was replaced by two cycles of 30 s (with a 10 s break in between) in the Bead Ruptor Elite at a speed of 5.5 m/s. Additionally, two negative controls in which the sample material was replaced by sterile, DNA-free water was included to confirm absence of reagent contamination. DNA samples were then subjected to PCR amplification of the hypervariable region of the bacterial 16S rRNA gene (primers 515 F and 806 R) [75] and the fungal ITS1 region (primers BITS and B58S3) [76] using Illumina barcoded primers, designed according to Kozich et al. (2013) [75] (dual-index sequencing strategy; Additional file 1: Table S11 and S12). In each run, two negative PCR controls (in which DNA template was replaced by DNA-free water) and a DNA mock community sample (one for bacteria and one for fungi) were included. Both mock communities were composed of a number of species that were likely to occur in insects [29] (Additional file 1: Table S13). PCR amplification was performed in a reaction volume of 40 µL, consisting of 2 µL DNA, 0.5 µM of each primer, 150 µM of each dNTP, 1 × Titanium Taq PCR buffer and 1 × Titanium Taq DNA polymerase (Takara Bio, Saint-Germain-en-Laye, France). The reactions were initiated by denaturation at 94 °C for 120 s, followed by 35 cycles of 45 s at 95 °C, 45 s at 59 °C and 45 s at 72 °C, and a final elongation step of 10 min at 72 °C. All samples were successfully amplified for bacteria, while fungal amplicons were only obtained for 273 out of 289 samples. For the negative DNA extraction and PCR controls, very faint to no bands were obtained after gel electrophoresis. Amplicons from positive insect samples as well as from the different controls were purified using Agencourt AMPure XP magnetic beads (Beckman Coulter Genomics GmbH, South Plainfield, UK) following the manufacturer’s instructions. Subsequently, a Qubit high sensitivity fluorometer (Invitrogen, Carlsbad, USA) was used to measure the concentration of the purified amplicons, and each sample was then pooled in equimolar concentrations into two libraries, one pool of bacterial V4 amplicons and one pool of fungal ITS1 amplicons. Next, following ethanol precipitation, the amplicon pools were loaded onto a 1.5% agarose gel, and the bands corresponding to the expected fragment length were excised from the gel and purified using a QIAquick Gel Extraction Kit (Qiagen, Hilden, Germany). Following gel extraction, the concentration of the libraries was measured again, diluted to 2 nM, and then sent for sequencing at the Center for Medical Genetics (University of Antwerp, Antwerp, Belgium) using an Illumina MiSeq sequencer with a v2 500-cycle reagent kit (Illumina, San Diego, USA). Additionally, for a subset of randomly selected samples (10 per group), bacterial and fungal densities were quantified by determining total bacterial 16S rRNA gene and fungal ITS1 copy numbers using qPCR with the same primers pairs as those used for the sequencing approach (but without barcodes) (for details, see [77]). Furthermore, the same samples were subjected to a PCR analysis targeting the 16S rRNA gene of *Wolbachia* (wspec primers) as previously described [78]. Both PCR analyses were performed in duplicate.

Illumina sequences were received as a demultiplexed FASTQ file, with barcodes and primer sequences removed. For the V4 sequences, paired-end reads were merged using USEARCH (v11.0.667) to form consensus sequences [79] with not more than 10 mismatches allowed in the overlap region. For the ITS sequences, only forward reads were retained. Subsequently, sequences were truncated at the 248th base, and reads shorter than 248 bp or reads with a total expected error threshold above 0.2 and 1 for the V4 and ITS regions, respectively, were discarded using USEARCH (v11.0.667). Next, Mothur’s (v1.39.3) commands ‘classify.seqs’ and ‘remove.lineage’ or ‘get.lineage’ in combination with the Silva database (v1.38, for bacteria) and UNITE database (v6, for fungi), respectively, were used to identify and remove potential mitochondrial, chloroplast or other non-target sequences. Bacterial sequences were classified into zero-radius operational taxonomic units (zOTUs [80]; also known as amplicon sequence variants (ASVs) [81]) by the UNOISE3 algorithm as implemented in USEARCH [82]. Only zOTUs with a minimum abundance of eight reads were kept and chimeric sequences identified by the algorithm were removed. Fungal sequences were clustered into operational taxonomic units (OTUs) based on a 3% sequence dissimilarity cut-off. The advantage of zOTUs is that they enable resolution of closely related taxa that would be incorporated into the same OTU when applying a 3% dissimilarity cut-off. However, given that many fungal species house intraspecific and intragenomic variations in ITS1 [83] fungal diversity is still commonly assessed by the use of 97% OTUs as fungal species proxies [84]. OTU clustering was performed using the UPARSE greedy algorithm in USEARCH, during which chimeric sequences were also removed [79], as were global singletons (i.e. OTUs with only 1 sequence represented in the entire data set). Next, both the bacterial and fungal data sets were analyzed in R (v3.5.2) using microDecon (v1.2.0) [85] to control for the presence of contaminants based on (z)OTU prevalence in the insect samples versus the mean of the two PCR control samples [86, 87]. At the same time, the DNA extraction controls were removed from the dataset since they yielded only very low sequence numbers (less than 100). Additionally, (z)OTUs occurring below a 0.1% and OTUs occurring below 1% relative abundance threshold per sample were discarded from further analysis (which was in accordance with the thresholds defined by the mocks communities). Finally, the number of sequences was rarefied to 2500 sequenced for bacteria and 1000 sequences for fungi. The taxonomic origin of each bacterial zOTU and fungal OTU was determined with the SINTAX algorithm as implemented in USEARCH based on the SILVA Living Tree Project v123 for bacteria and the UNITE database v6 for fungi. Further, the identity of the most important zOTUs and OTUs was verified with a BLAST search in GenBank against type materials. When no significant similarity was found with type materials (< 97% identity), the BLAST analysis was performed against entire GenBank. For fungi, for which less type strain sequences are available, the BLAST search was performed against both type strains and the GenBank database excluding uncultured and environmental sample sequences. Analysis of the mock communities demonstrated that only the expected taxa were found, indicating that the experimental conditions were met to achieve robust data.

### Data analysis

For each sample, a rarefaction curve was generated to see whether (z)OTU richness reached an asymptote. Rarefaction curves were created using the Phyloseq package in R showing the number of observed (z)OTUs as a function of the number of sequences [87, 88]. Subsequently, observed (z)OTU richness and Shannon diversity were calculated for each sample with the phyloseq package in R. The Shannon diversity index is an index that is commonly used to characterize species diversity in a community and accounts for both abundance and evenness of the species present. Higher scores indicate high diversity, while scores close to 0 indicate low diversity [89]. Samples were grouped according to habitat (field-collected vs. lab-reared caterpillars), organism (caterpillar vs. parasitoid larvae), origin (external vs. internal) and health status (healthy vs. parasitized). For the caterpillars, a three-way analysis of variance (ANOVA) was used to assess whether habitat, origin and health status affected species richness, Shannon diversity and microbial densities. All two-way interactions and three-way interaction were included in the model as well. Based on the Hellinger transformed relative abundance data of the observed bacteria and fungi in each of the sampled individuals, the bacterial and fungal community composition was visualized by non‐metric multidimensional scaling (NMDS) using the Bray–Curtis coefficient as distance measure in the R software package vegan [90]. To test the hypothesis that caterpillars bacterial and fungal communities differed between habitats, origin and health status, permutational analysis of variance (PERMANOVA) [91] was performed using the “adonis” function in the software package vegan [90]. All factors and their interactions were included as fixed factors in the analysis. Significance was tested using 1,000 permutations. All analyses were performed for bacteria and fungi separately.

## Supplementary Information

**Additional file 1: Table S1.** zOTUs bacteria. Identification of bacterial zero radius operational taxonomic units (ZOTUs) according to the Silva v1.23 database and distribution over the investigated samples. **Table S2.** OTUs fungi. Identification of fungal operational taxonomic units (OTUs) according to the UNITE v6 database and distribution over the investigated samples. **Table S3.** 3 way ANOVA. Results of three way ANOVA of observed richness, Shannon diversity and densities for bacteria and fungi (caterpillars only). **Table S4.** Diversity bacteria. Diversity metrics for the bacterial communities. **Table S5.** Diversity fungi. Diversity metrics for the fungal communities. **Table S6.** Copy number bacteria. Determination of bacterial 16S rRNA gene copy numbers using qPCR. **Table S7.** Copy number fungi. Determination of fungal ITS copy numbers using qPCR. **Table S8.** *Wolbachia* detection. Occurrence of *Wolbachia* in studied samples, as determined using a specific *Wolbachia* PCR. **Table S9.** Plating results. Plating results: log number of colony forming units (cfu) per caterpillar (*n* = 2 per subgroup of health status). **Table S10.** Sample details. Sample details, including number of caterpillars used in this study. **Table S11.** Illumina primers V4. Primer design and sample-specific barcodes for the bacterial V4 region of the 16S rRNA gene. **Table S12.** Illumina primers ITS. Primer design and sample-specific barcodes for the fungal ITS region. **Table S13.** Mocks. Composition of mock communities.

**Additional file 2: Fig. S1.** Rarefaction curves for the different samples studied, based on the bacterial V4 dataset (A) and the fungal ITS dataset (B). Rarefaction curves approached saturation, indicating that our sequencing depth was sufficient to cover the microbial diversity. **Fig. S2.** Fungal community profiles of the different caterpillars (*Pieris brassicae*) and parasitoid larvae (*Cotesia glomerata*) samples studied. Fungal taxa represent the most prevalent taxa in the different subgroups based on origin and health status for caterpillars and origin for parasitoid larvae (present at a mean relative abundance > 1% in at least one subgroup). For each OTU, the average relative abundance for each subgroup is given in the box as a percentage, whereas the color indicates prevalence (white is absent). OTUs are identified by a BLAST search against GenBank excluding uncultured/environmental sample sequences. Identifications were performed at genus level; when identical scores were obtained for different genera, identifications were performed at a higher taxonomic level. When identity percentages were lower than 99%, the percentage of sequence identity with the GenBank entry is given between brackets. Abbreviations used: H = healthy; P = parasitized; E = external; and I = internal.

## Data Availability

The sequences obtained in this study were deposited in the Sequence Read Archive (SRA) at NCBI under Bioproject PRJNA700075.
