# Supplementary material for: Parasitism by endoparasitoid wasps alters the internal but not the external microbiome in host caterpillars
Source: Anim Microbiome. 2021 Oct 15;3:73. doi: 10.1186/s42523-021-00135-y (PMC8520287; doi:10.1186/s42523-021-00135-y)
Supplement: Supplementary file 2 — Additional file 2: Fig. S1. Rarefaction curves for the different samples studied, based on the bacterial V4 dataset (A) and the fungal ITS dataset (B). Rarefaction curves approached saturation, indicating that our sequencing depth was sufficient to cover the microbial diversity. Fig. S2. Fungal community profiles of the different caterpillars (Pieris brassicae) and parasitoid larvae (Cotesia glomerata) samples studied. Fungal taxa represent the most prevalent taxa in the different subgroups based on origin and health status for caterpillars and origin for parasitoid larvae (present at a mean relative abundance > 1% in at least one subgroup). For each OTU, the average relative abundance for each subgroup is given in the box as a percentage, whereas the color indicates prevalence (white is absent). OTUs are identified by a BLAST search against GenBank excluding uncultured/environmental sample sequences. Identifications were performed at genus level; when identical scores were obtained for different genera, identifications were performed at a higher taxonomic level. When identity percentages were lower than 99%, the percentage of sequence identity with the GenBank entry is given between brackets. Abbreviations used: H = healthy; P = parasitized; E = external; and I = internal. [file 42523_2021_135_MOESM2_ESM.pdf]

Figure S1

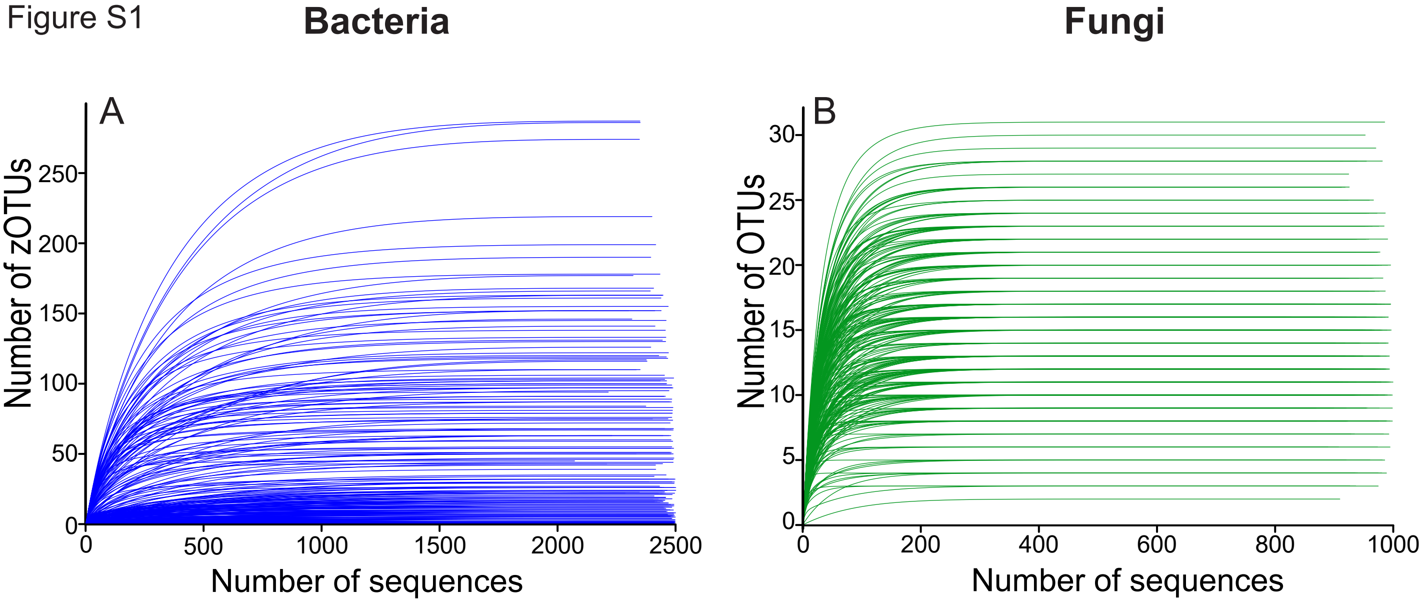
Bacteria
Fungi
A
Number of zOTUs
250
200
150
100
50
0
0
500
1000
1500
2000
2500
Number of sequences
B
Number of OTUs
30
25
20
15
10
5
0
0
200
400
600
800
1000
Number of sequences

Figure S2

	Field-collected						Lab-reared					
	Caterpillars				Larvae		Caterpillars				Larvae	
	H-E	P-E	H-I	P-I	E	I	H-E	P-E	H-I	P-I	E	I
Alternaria	13.3	9.5	6.3	9.2	10.0	7.2	26.5	22.5	13.5	9.8	3.2	5.7
Alternaria	24.7	17.6	24.7	12.0	9.8	6.6	3.3	4.8	7.1	7.4	5.2	7.6
Sporobolomyces	14.2	19.7	11.6	13.0	5.5	5.9	1.0	2.6	3.2	6.6	6.3	10.1
Exophiala			<1.0	1.8	1.0	4.8	<1.0	<1.0	<1.0	<1.0	2.0	<1.0
Mucor	2.6	1.3	4.2	5.2	2.8	3.1	<1.0	<1.0	1.7	1.4	1.4	1.1
Cladosporium	2.5	3.1	2.8	1.6	<1.0	1.8	7.7	1.8	4.6	<1.0		<1.0
Malassezia	<1.0	<1.0	1.4	1.2	3.3	3.9	<1.0	4.0	1.7	7.6	5.7	3.7
Debaryomyces	1.2	<1.0	<1.0	1.6	3.5	3.9	1.1	1.3	1.1	3.1	3.0	2.0
Xylodon	<1.0	<1.0	<1.0	<1.0	<1.0	2.1	<1.0	<1.0	<1.0	2.0	2.8	4.1
Trametes	<1.0	<1.0	<1.0	<1.0	1.9	3.5	<1.0	<1.0	1.1	2.5	2.8	4.1
Stemphylium	1.3	1.3	<1.0	<1.0	<1.0		2.4	<1.0	5.1	<1.0	<1.0	<1.0
Aureobasidium	1.3	2.5	<1.0	<1.0	<1.0	<1.0	<1.0	<1.0	<1.0	<1.0	<1.0	<1.0
Geosmithia	<1.0		<1.0		1.2	1.2	5.1	1.5	2.5	1.5	1.0	<1.0
Filobasidium	2.7	4.4	2.2	2.4	1.4	1.4	<1.0	1.1	<1.0	1.5	1.5	2.2
Dissoconium (98.8%)				1.6			<1.0	<1.0	<1.0			
Cystofilobasidium	<1.0	8.2	2.3	4.5	2.1	<1.0	<1.0	<1.0	2.0	1.5	1.2	3.9
Ganoderma	<1.0	<1.0	<1.0	<1.0	1.1	<1.0	<1.0		<1.0	<1.0	<1.0	
Clitocybe	<1.0	<1.0	<1.0	<1.0	4.1	<1.0	<1.0	<1.0	<1.0	<1.0	<1.0	<1.0
Pleosporales			<1.0		2.4	<1.0						<1.0
Pyrenophora	1.3	2.1	1.2	<1.0	<1.0		<1.0		<1.0		<1.0	<1.0
Alternaria	<1.0	1.2	<1.0	1.2	<1.0	<1.0	<1.0	<1.0	<1.0	<1.0	<1.0	<1.0
Aspergillus			<1.0		<1.0	<1.0	1.5	1.0	3.0	1.0	<1.0	
Gibellulopsis	1.4	<1.0	1.1	2.4	1.4	<1.0	2.3	4.8	4.1	1.2	<1.0	1.6
Armillaria				<1.0	<1.0	1.2	<1.0	5.2		<1.0		
Plicaturopsis				<1.0	<1.0	<1.0	<1.0	3.9	<1.0	2.6	<1.0	<1.0
Peniophora	<1.0	<1.0	<1.0	<1.0	1.1	<1.0	<1.0	<1.0	<1.0	<1.0		<1.0
Alternaria	<1.0	<1.0	1.1	<1.0								
Pyrenophora	1.7	<1.0		<1.0		<1.0						
Filobasidium	2.1	2.3	1.1	1.0	<1.0	1.1		1.1	<1.0	<1.0	2.1	<1.0
Thelebolus	1.1		<1.0	<1.0	<1.0			<1.0			<1.0	
Vishniacozyma (98.7%)	1.1	2.0	<1.0	2.4	1.0	1.4	<1.0	<1.0	<1.0	1.4	1.2	2.0
Alternaria	<1.0		<1.0	<1.0	1.5	<1.0		<1.0	<1.0	<1.0	<1.0	<1.0
Neodevriesia				1.9								
Aspergillus	<1.0		1.7	<1.0	3.8	2.6	1.1	1.2	1.7	<1.0	4.1	<1.0
Cladosporium					<1.0	<1.0	2.8	<1.0	1.4	<1.0		<1.0
Itersonilia	<1.0	<1.0	1.3	1.3	2.4	<1.0	<1.0	1.2	<1.0	1.5	1.5	5.2
Epicoccum	2.0	1.1	1.8	1.4	<1.0	1.0	2.9	2.5	<1.0	<1.0	<1.0	<1.0
Aspergillus					<1.0				<1.0	5.6	<1.0	
(Globi)fomes	<1.0	<1.0	<1.0	<1.0	<1.0	<1.0				<1.0	1.0	<1.0
Resinicium	<1.0		<1.0		<1.0		<1.0		<1.0	<1.0	1.6	<1.0
Gymnostellatospora		<1.0	<1.0	<1.0	1.0	<1.0	<1.0	<1.0	<1.0	1.1		<1.0
Exophiala			<1.0	1.1	<1.0	<1.0		<1.0	<1.0	<1.0		
Dothideomycetes	1.7								<1.0			
Mucor		<1.0		1.3		<1.0		<1.0				
Hypocreales	<1.0	<1.0		<1.0	<1.0	1.8	<1.0	1.3	<1.0	<1.0	<1.0	<1.0
Ceriporiopsis							1.3		<1.0			
Puccinia	<1.0	<1.0	1.0	2.3		<1.0	<1.0	<1.0	<1.0			
Tremellaceae (98.7%)	<1.0	<1.0	1.7	1.2	<1.0	<1.0		<1.0	<1.0	<1.0	<1.0	1.1
Cystofilobasidium											4.5	
Filobasidium	<1.0	1.7	<1.0	<1.0	<1.0	<1.0	<1.0	<1.0		<1.0	<1.0	<1.0
Plectosphaerella (98.8%)	<1.0	<1.0	<1.0	<1.0	<1.0	<1.0	1.4			<1.0	<1.0	<1.0
Peniophora	<1.0	<1.0		<1.0	<1.0	<1.0	<1.0	1.2	<1.0	1.4	1.3	<1.0
Malassezia			<1.0	<1.0	2.5		<1.0					
Fusarium (94.4%)	1.0	1.9	<1.0	<1.0		<1.0	<1.0			<1.0		
Polyporus								2.5		<1.0		
Xylodon		<1.0					<1.0			1.9		<1.0
Hyphodontia				1.3		<1.0						
Acremonium			<1.0				1.2		1.2			1.7
Meyerozyma					<1.0	<1.0	<1.0	<1.0	<1.0	<1.0		1.1
Gibellulopsis (98.8%)	1.0	<1.0	<1.0	<1.0	1.0	<1.0	<1.0	2.8	<1.0	<1.0	<1.0	<1.0
Malassezia	<1.0	<1.0		<1.0				<1.0	<1.0	<1.0		1.1
Itersonilia	<1.0	<1.0	<1.0	<1.0	<1.0	<1.0	<1.0	<1.0	1.0	1.6	<1.0	<1.0
Melampsora	<1.0	<1.0							1.0		<1.0	1.2
Naganishia	<1.0		<1.0	<1.0	1.1	<1.0				<1.0		1.0
Rhodotorula	<1.0				<1.0	<1.0	1.6	<1.0	<1.0	<1.0	3.5	<1.0
Rhodotorula						<1.0	<1.0	<1.0	2.5			
Heterobasidion			<1.0								1.9	
Anthracocystis							<1.0	1.3	<1.0			<1.0
Mucor	<1.0		<1.0				2.9	2.6	2.5	<1.0		
Physcia		<1.0		<1.0		<1.0	<1.0			1.6		<1.0
Taphrina	<1.0							1.1	<1.0	<1.0		
Pleosporales		<1.0	<1.0		1.3	<1.0		<1.0		<1.0	<1.0	<1.0
Cystofilobasidium	<1.0	1.6	<1.0	<1.0	<1.0				<1.0	<1.0		
Fusarium	2.1	<1.0	<1.0									<1.0
Cyberlindnera						2.5			<1.0			
Postia						1.3						
Dioszegia	<1.0			1.5								
Beauveria	<1.0							<1.0	<1.0		<1.0	1.5
Aspergillus	<1.0		2.0		<1.0							
Cystobasidium			<1.0		<1.0	<1.0	<1.0			1.2	<1.0	
Phialocephala (98.2%)								1.8				
Cystobasidium						<1.0	<1.0	<1.0	1.3		<1.0	
Phaeosphaeria			1.3									
Exidia (98.8%)							<1.0					1.0
Fomes		<1.0							<1.0	1.3	2.2	1.9
Penicillium (98.8%)			<1.0		<1.0	<1.0	1.3	1.5	1.8	1.2	<1.0	<1.0

Prevalence (%)
100 80 60 40 20 0
